# Music and speech prosody: a common rhythm

**DOI:** 10.3389/fpsyg.2013.00566

**Published:** 2013-09-02

**Authors:** Maija Hausen, Ritva Torppa, Viljami R. Salmela, Martti Vainio, Teppo Särkämö

**Affiliations:** ^1^Cognitive Brain Research Unit, Institute of Behavioural Sciences, University of HelsinkiHelsinki, Finland; ^2^Finnish Center of Excellence in Interdisciplinary Music Research, University of JyväskyläJyväskylä, Finland; ^3^Institute of Behavioural Sciences, University of HelsinkiHelsinki, Finland; ^4^Department of Speech Sciences, Institute of Behavioural Sciences, University of HelsinkiHelsinki, Finland

**Keywords:** music perception, MBEA, speech prosody perception, word stress, visuospatial perception

## Abstract

Disorders of music and speech perception, known as amusia and aphasia, have traditionally been regarded as dissociated deficits based on studies of brain damaged patients. This has been taken as evidence that music and speech are perceived by largely separate and independent networks in the brain. However, recent studies of congenital amusia have broadened this view by showing that the deficit is associated with problems in perceiving speech prosody, especially intonation and emotional prosody. In the present study the association between the perception of music and speech prosody was investigated with healthy Finnish adults (*n* = 61) using an on-line music perception test including the Scale subtest of Montreal Battery of Evaluation of Amusia (MBEA) and Off-Beat and Out-of-key tasks as well as a prosodic verbal task that measures the perception of word stress. Regression analyses showed that there was a clear association between prosody perception and music perception, especially in the domain of rhythm perception. This association was evident after controlling for music education, age, pitch perception, visuospatial perception, and working memory. Pitch perception was significantly associated with music perception but not with prosody perception. The association between music perception and visuospatial perception (measured using analogous tasks) was less clear. Overall, the pattern of results indicates that there is a robust link between music and speech perception and that this link can be mediated by rhythmic cues (time and stress).

## Introduction

Music and speech have been considered as two aspects of the highly developed human cognition. But how much do they have in common? Evolutionary theories suggest that music and speech may have had a common origin in form of an early communication system based on holistic vocalizations and body gestures (Mithen, 2005) and that music may have played a crucial role in social interaction and communication, especially between the mother and the infant (Trehub, 2003). Another view holds that the development of music can be understood more as a by-product of other adaptive functions related to, for example, language, and emotion (Pinker, 1997). Whether their origins are linked or not, both music and speech are auditory communication systems that utilize similar acoustic cues for many purposes, for example for expressing emotions (Juslin and Laukka, 2003). Especially in infant-directed speech, the musical aspects of language (rhythm, timbral contrast, melodic contour) are the central means of communication, and there is novel evidence that newborns show largely overlapping neural activity to infant-directed speech and to instrumental music (Kotilahti et al., 2010). It has been suggested that the musical aspects of language might also be used as scaffolding for the later development of semantic and syntactic aspects of language (Brandt et al., 2012).

In addition to the links that have been found in early development, music and speech seem to be behaviorally and neurally interrelated also later in life. Evidence from functional resonance imaging (fMRI) studies of healthy adults suggests that perceiving music and speech engages at least partly overlapping neural regions, especially in superior, anterior and posterior temporal areas, temporoparietal areas, and inferior frontal areas (Koelsch et al., 2002; Tillmann et al., 2003; Rauschecker and Scott, 2009; Schön et al., 2010; Abrams et al., 2011; Rogalsky et al., 2011), including also Broca's and Wernicke's areas in the left hemisphere that were previously thought to be language-specific. Similarly, studies using electroencephalography (EEG) and magnetoencephalography (MEG) have shown that in both speech and music the discrimination of phrases induces similar closure positive shift (CPS) responses (Steinhauer et al., 1999; Knösche et al., 2005) and that syntactic violations in both speech and music elicit similar P600 responses in the brain (Patel et al., 1998). An EEG study of healthy non-musicians also showed that music may induce similar semantic priming effects as words when semantically related or unrelated words are presented visually after hearing music excerpts or spoken sentences (Koelsch et al., 2004).

A clear link between speech and music has also been shown in behavioral and neuroimaging studies of musical training (for a recent review, see Kraus and Chandrasekaran, 2010; Shahin, 2011). Compared to non-musicians, superior speech processing skills have been found in adult musicians (Schön et al., 2004; Chartrand and Belin, 2006; Marques et al., 2007; Lima and Castro, 2011; Marie et al., 2011a,b) and musician children (Magne et al., 2006). Also, musical training has been shown to enhance speech-related skills in longitudinal studies where the non-musician participants were randomly assigned to a music training group and a control group (Thompson et al., 2004; Moreno et al., 2009; Dege and Schwarzer, 2011; Chobert et al., 2012; François et al., 2012). The superior speech-related skills of musicians or participants in musical training group include the perception of basic acoustic cues in speech, such as pitch (Schön et al., 2004; Magne et al., 2006; Marques et al., 2007; Moreno et al., 2009), timbre (Chartrand and Belin, 2006), and vowel duration (Chobert et al., 2012). These results support the hypothesis that music and speech are at least partly based on shared neural resources (Patel, 2008, 2012). The improved processing of these basic acoustic parameters can also lead to enhanced processing of more complex attributes of speech, which can be taken as evidence of transfer of training effects (Besson et al., 2011). The enhanced higher level processing of speech includes speech segmentation (Dege and Schwarzer, 2011; François et al., 2012) and the perception of phonemic structure (Dege and Schwarzer, 2011), metric structure (Marie et al., 2011b), segmental and tone variations in a foreign tone-language (Marie et al., 2011a), phonological variations (Slevc and Miyake, 2006) and emotional prosody (Thompson et al., 2004; Lima and Castro, 2011). Musical ability is also related to enhanced expressive language skills, such as productive phonological ability (Slevc and Miyake, 2006) and pronunciation (Milovanov et al., 2008) in a foreign language, as well as reading phonologically complex words in one's native language (Moreno et al., 2009).

The enhanced processing of linguistic sounds is coupled with electrophysiologically measured changes across different auditory processing stages, starting from the brainstem (Musacchia et al., 2007; Wong et al., 2007) and extending to the auditory cortex and other auditory temporal lobe areas (Magne et al., 2006; Musacchia et al., 2008; Moreno et al., 2009; Marie et al., 2011a,b). Years of musical training have been found to correlate with stronger neural activity induced by linguistic sounds at both subcortical and cortical levels (Musacchia et al., 2008). This result and the results of the longitudinal studies (Thompson et al., 2004; Moreno et al., 2009; Dege and Schwarzer, 2011; Chobert et al., 2012; François et al., 2012) suggest that the possible transfer effects are more likely results of training rather than genetic predispositions. When studying the possible transfer of training effects of music expertise on speech processing, it is important to consider general cognitive abilities as possible mediators. ERP studies show that attention does not explain the effects, but results regarding memory present a more mixed picture (Besson et al., 2011). A clear correlation between music lessons and general intelligence has been found (Schellenberg, 2006), indicating that the transfer effects between music and language can partly be explained by enhanced general cognitive abilities when not controlled.

Conversely, language experience may also have an effect on the development of music perception. For example, speakers of a tone-language (e.g., Chinese) have better abilities in imitating and discriminating musical pitch (Pfordresher and Brown, 2009; Bidelman et al., 2011) and they acquire absolute pitch more often than Western speakers (Deutsch et al., 2006). Also, speakers of a quantity language (Finnish) have been found to have similar enhanced processing of duration of non-speech sounds as French musicians, compared to French non-musicians (Marie et al., 2012).

Processing speech and music appear to be linked in the healthy brain, but does the same hold true in the damaged brain? Disorders of music and speech perception/expression, known as amusia and aphasia, have traditionally been regarded as independent, separable deficits based on double dissociations observed in studies of brain damaged patients (amusia without aphasia: Peretz, 1990; Peretz and Kolinsky, 1993; Griffiths et al., 1997; Dalla Bella and Peretz, 1999; aphasia without amusia: Basso and Capitani, 1985; Mendez, 2001; for a review, see Peretz and Coltheart, 2003). However, recent studies suggest that this double dissociation may not be absolute. In Broca's aphasia, problems in the syntactic (structural) processing of language have been shown to be associated with problems in processing structural relations in music (Patel, 2005; Patel et al., 2008a). Musical practices are useful also in the rehabilitation of language abilities of patients with non-fluent aphasia (Racette et al., 2006; Schlaug et al., 2010; Stahl et al., 2011), suggesting a further link between the processing of speech and music in the damaged brain. Moreover, persons with congenital amusia have been found to have lower than average abilities in phonemic and phonological awareness (Jones et al., 2009), in the perception of emotional prosody (Thompson, 2007; Thompson et al., 2012), speech intonation (Patel et al., 2005, 2008b; Jiang et al., 2010; Liu et al., 2010) and subtle pitch variation in speech signals (Tillmann et al., 2011b), and in the discrimination of lexical tones (Nan et al., 2010; Tillmann et al., 2011a). Collectively, these results suggest that amusia may be associated with fine-grained deficits in the processing of speech.

Similar to music, the central elements in speech prosody are melody (intonation) and rhythm (stress and timing) (Nooteboom, 1997). Studies of acquired amusia show that the melodic and rhythmic processing of music can be dissociated (Peretz, 1990; Peretz and Kolinsky, 1993; Di Pietro et al., 2004), suggesting that they may be partly separate functions. Previously, the association between music and speech processing has mainly been found to exist between the perception of the melodic aspect of music and speech (Schön et al., 2004; Patel et al., 2005, 2008b; Magne et al., 2006; Marques et al., 2007; Moreno et al., 2009; Jiang et al., 2010; Liu et al., 2010; Nan et al., 2010). However, also rhythm has important functions in both music and speech. Speech is perceived as a sequence of time, and the term speech rhythm is used to refer to the way these events are distributed in time. The patterns of stressed (strong) and unstressed (weak) tones or syllables build up the meter of both music and speech (Jusczyk et al., 1999; for a review, see Cason and Schön, 2012). Speech rhythm can be used in segmenting words from fluent speech: the word stress patterns that are typical in one's native language help to detect word boundaries (Vroomen et al., 1998; Houston et al., 2004). Depending on the language, word stress is expressed with changes in fundamental frequency, intensity, and/or duration (Morton and Jassem, 1965). Fundamental frequency (f0) is often thought to be a dominant prosodic cue for word stress (Lieberman, 1960; Morton and Jassem, 1965) and word segmentation (Spinelli et al., 2010)—however, changes in syllable duration and sound intensity are also associated with the prosodic patterns that signal stress (Lieberman, 1960; Morton and Jassem, 1965). For example, the results from Kochanski et al. (2005) suggest that in English intensity and duration may play even more important role for the detection of syllabic stress than f0. In Finnish, word or lexical stress alone is signaled with durational cues (Suomi et al., 2003), as well as intensity, whereas sentence stress is additionally signaled with fundamental frequency (Vainio and Järvikivi, 2007).

Although there are relatively few studies looking at rhythm or meter associating the perception of music and speech, there are some recent findings that support this association. For example, Marie et al. (2011b) found that musicians perceive the metric structure of words more accurately than non-musicians: incongruous syllable lengthenings elicited stronger ERP activations in musicians both automatically and when it was task-relevant. Also, priming with rhythmic tones can enhance the phonological processing of speech (Cason and Schön, 2012) and the synchronizing of musical meter and linguistic stress in songs can enhance the processing of both lyrics and musical meter (Gordon et al., 2011).

Another cognitive domain that has recently been linked to music perception is visuospatial processing. A stimulus-response compatibility effect has been found between the pitch (high/low) of auditory stimuli and the location (up/down) of the answer button (Rusconi et al., 2006). There is also evidence that musicians' abilities in visuospatial perception are superior to average (Brochard et al., 2004; Patston et al., 2006). Moreover, congenital amusics have been found to have below average performance in a mental rotation task (Douglas and Bilkey, 2007), although this finding has not been replicated (Tillmann et al., 2010). Williamson et al. (2011) found that a subgroup of amusics were slower but as accurate as the control group in the mental rotation task, but did not find any group differences in a range of other visuospatial tasks. Douglas and Bilkey (2007) also found that the stimulus-response compatibility effect was not as strong in amusics as in the control group. In another study, the amusic group reported more problems in visuospatial perception than the control group, but this was not confirmed by any objective measure (Peretz et al., 2008). Taken together, there is some preliminary evidence that visuospatial and musical processing might be linked, but more research is still clearly needed.

The main aim of the present study was to systematically determine the association between music perception (as indicated by a computerized music perception test including the Scale subtest of the Montreal Battery of Evaluation of Amusia, as well as Off-beat and Out-of-key tasks) and the perception of speech prosody, using a large sample of healthy adult subjects (*N* = 61). To measure the perception of speech prosody, we used a novel experiment that does not focus only on pitch contour (such as the statement-question sentence tests used in many previous studies) but measures the perception of word stress utilizing a natural combination of fundamental frequency, timing and intensity variations. Thus, this experiment is suitable for assessing the possible connection of perception of both rhythm and pitch in music to prosodic perception. We concentrate on the role of the acoustic differences in the perception of word stress, not the linguistic aspects of this prosodic phenomenon (see, for example, Vogel and Raimy, 2002). Second, the study investigated the possible association between visuospatial perception and music perception. Possible confounding variables, including auditory working memory and pitch perception threshold, were controlled for.

## Materials and methods

### Participants

Sixty four healthy Finnish adults were recruited into the study between June and August 2011. The ethical committee of the Faculty of Behavioural Sciences of the University of Helsinki approved the study and the participants gave their written informed consent. Inclusion criteria were age between 19–60 years, self-reported normal hearing and speaking Finnish as first language or at a comparable level (by self-report). Exclusion criteria were being a professional musician and/or having obtained music education at a professional level. From the 64 tested participants, 13 reported having visited an audiologist—one participant was excluded from the analysis because of a deaf ear. However, the other participants who had visited an audiologist had suspected hearing problems that had proved to be either non-existent, transient, or very mild (reported by the participants and controlled by statistical analyses, see section Associations Between the Music Perception Test and Demographical and Musical Background Variables). None of the participants had a cerebral vascular accident or a brain trauma. Another participant was excluded because of weaker than first language level skills in Finnish. One participant was found to perform significantly (>3 SD) below the average total score in the music perception test. In questionnaires, this participant also reported “lacking sense of music” and “being unable to discriminate out-of-key tones,” further suggesting that the participant might have congenital amusia. In order to limit this study to healthy participants with musical abilities in the “normal” range (without musical deficits or professional expertise in music), the data from this participant was excluded from further analysis. Thus, data from 61 participants was used in the statistical analysis. Fifty-eight (95.1%) of the analyzed participants spoke Finnish as their first language and three participants (4.9%) spoke Finnish at a level comparable to first language. Other characteristics of the analyzed participants are shown in Table 1.

**Table 1:** **Characteristics of the participants**.

Male/female	21/40 (34/66%)
Mean age (range)	39.0 (19–59)
Education Level	
Primary level	0 (0%)
Secondary level	23 (38%)
Lowest level tertiary	6 (10%)
Bachelor level	17 (28%)
Master level or higher	15 (25%)
Mean education in years (range)	17.1 (10–32)
Musical education: no/yes	19/42 (31/69%)
Musical playschool	4 (7%)
Special music class in school	6 (10%)
Private lessons or with parents	23 (37%)
Music institute or conservatory	13 (21%)
Independent music learning	26 (43%)
Mean musical training in years (range)	3.7 (0–19)
Self-reported cognitive problems	
Reading problems	5 (8%)
Speech problems	3 (5%)
Spatial orientation problems	5 (8%)
Problems in maths	12 (20%)
Attentional problems	5 (8%)
Memory problems	6 (10%)

### Assessment methods

Music, speech prosody, pitch, and visuospatial perception abilities were assessed with computerized tests and working memory was evaluated using a traditional paper-pencil test. The computer was a laptop with display size 12” and headphones. In addition, the participants filled out a paper questionnaire. The place of the testing was arranged individually for each participant: most assessments were done in a quiet work space at a public library. The researcher gave verbal instructions to all tests except the on-line music perception test, in which the participant read the instructions from the laptop screen. The duration of the assessment session was ca. 1.5 h on average, ranging from 1 to 2 h.

#### Music perception

Music perception was measured with an on-line computer-based music perception test including the Scale subtest of the original Montreal Battery of Evaluation of Amusia (MBEA; Peretz et al., 2003) as well as the Off-beat and Out-of-key tasks from the on-line version of the test (Peretz et al., 2008). The on-line version is constructed to measure the same underlying constructs as the MBEA and it has a high correlation with the original MBEA that is administered in laboratory setting (Peretz et al., 2008). The instructions were translated to Finnish and Swedish for the present study. The test used in the present comprised the Scale subtest (Peretz et al., 2003), the Off-beat subtest (Peretz et al., 2008), and the Out-of-key subtest (Peretz et al., 2008) (see http://www.brams.umontreal.ca/amusia-demo/ for a demo in English or French). The test included 30 melodies composed for the MBEA (Peretz et al., 2003) following Western tonal-harmonic conventions. The Scale subtest comprised piano tones while the Off-beat and the Out-of-key subtests used 10 different timbres (e.g., piano, saxophone, and clarinet). In the Scale subtest, the participants were presented with 31 trials, including one “catch” trial that was not included in the statistical analysis. Each trial was a pair of melodies and the task was to judge if the melodies were similar or different. In half (15) of the trials the melodies were the same and in half (15) of the trials the second melody had an out-of-scale tone (on average, 4.3 semitones apart from the original pitch). In the Off-beat and Out-of-key subtests, the subjects were presented with 24 trials of which 12 were normal melodies and 12 were incongruous by having a time delay (Off-beat) or an out-of-scale tone (Out-of-key) on the first downbeat in the third bar of the four-bar melody. In the Off-beat subtest the task was to judge if the melody contained an unusual delay. The 12 incongruous trials had a silence of 5/7 of the beat duration (i.e., 357 ms) prior to a critical tone disrupting the local meter. In the Out-of-key subtest the task was to judge if the melody contained an out-of-tune tone. In the incongruous 12 trials the melody had a 500 ms long tone that was outside the key of the melody, sounding like a “wrong note.” The subtests were always presented in the same order (Scale, Off-beat, Out-of-key) and each subtest began with 2–4 examples of congruous and incongruous trials. The volume level was adjusted individually to a level that was clearly audibly to the subject. In the end the participants filled out an on-line questionnaire about their history and musical background (see Appendix for the questionnaire in English; the participants filled it in Finnish or Swedish). The whole test was completed in 20–30 min.

#### Speech prosody (word stress) perception

Speech prosody perception was assessed with a listening experiment that measures the identification of word stress as it is produced to separate a compound word into a phrase of two separate words. The task examines the perception of word and syllabic stress as it is used to signal either word level stress or moderate sentence stress and it is designed so that all prosodic cues, namely f0, intensity, and duration, play a role (O'Halpin, 2010). The word stress examined in this study differs from so-called lexical stress, where the stress pattern differentiates the meaning of two phonetically identical words from each other, as well as from the sentence level stress, where a word is accented or emphasized to contrast it with other words in the utterance. The task is designed to measure the perception of syllabic stress at the level which aids in separating words from the surrounding syllables.

**Figure 1:**
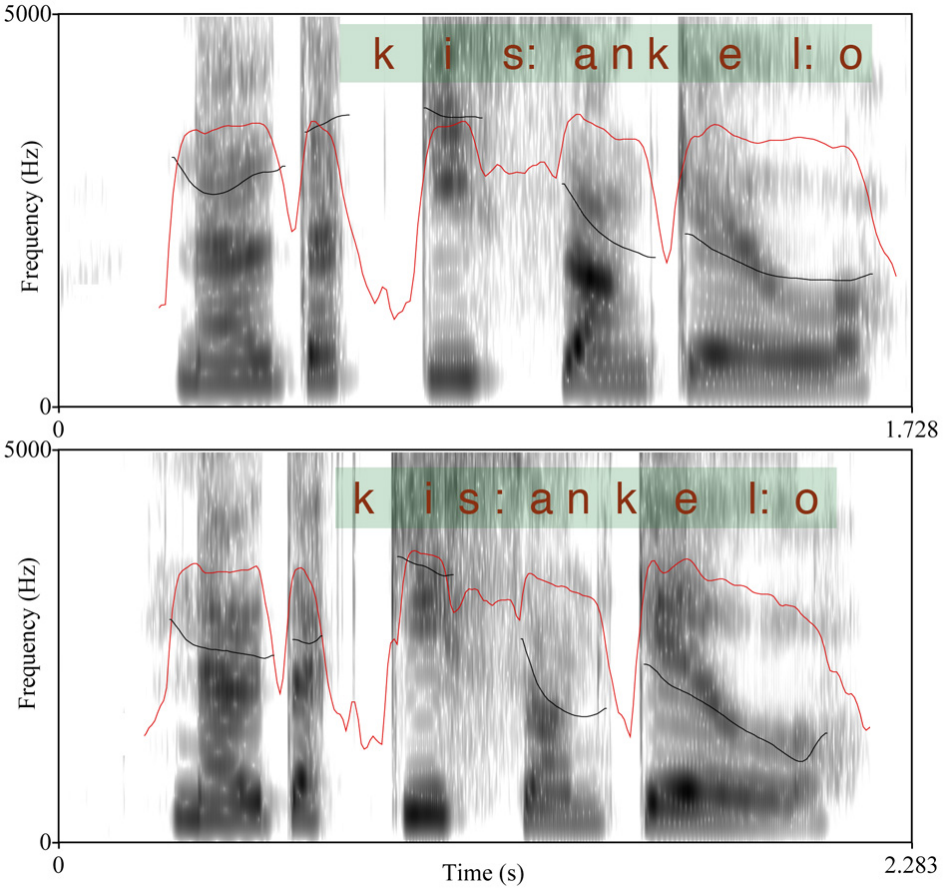
**Example of the spectrum of a compound word (above; audio file 1) and a two-word phrase (audio file 2) with f0 (black line) and intensity (red line) contours.** The scale is 9–400 Hz for f0 and 0–100 dB for intensity.

The test is originally based on work by Vogel and Raimy (2002) and O'Halpin (2010) and it has been adapted into Finnish by Torppa et al. (2010). Finnish has a fixed stress on the first syllable of a word; thus, a compound word has only one stressed syllable that is accented in an utterance context as opposed to two accents in a similar two word phrase. Typically, the first syllable of the second word of a compound has a secondary stress that differentiates it from a totally unstressed syllable. The materials in the test were spoken with a so called broad focus where (in the case of a phrase) neither of the two words stood out as more emphatic (as is the case in the so called narrow or contrastive focus). The stimuli were analyzed acoustically using Praat (Boersma, 2001) with respect to the (potentially) stressed syllables. We measured the raw f0 maxima, intensity maxima as well as the syllable durations and the differences between the values of the two syllables in each utterance were calculated; the results are summarized in Table 2. Table 2 shows the differences in f0, intensity, and duration between the first syllable of the first and second word of compound/phrased words and the results of paired *t*-tests on the significances of the differences. As shown in Table 2, for duration differences the statistical result did not reach significance—however, the differences between the compound vs. phrased utterances in the duration of the vowel (nucleus) in the second syllable of the second word was significant, *t*_(28)_ = −2.45, *p* = 0.02. Thus, the compound words were found to differ from the phrases with respect to all prosodic parameters (f0, duration, and intensity) showing that the difference was not produced with any single prosodic parameter. An example of an utterance pair (produced by a 10 year old female child) is shown in Figure 1. Each figure shows the spectrogram, f0 track, as well as intensity contour of the utterance. The extent of the words in question and the orthoghraphic text are also shown.

**Table 2:** **The differences between the cues for word stress in first and second stressed syllables in compound/phrase utterances**.

**Stimulus**	***N***	**Mean duration difference in ms (sd)**	**Mean f0 difference in semitones (sd)**	**Mean intensity difference in dB (sd)**
Compound	14	2.0 (69.2)	9.2 (2.4)	8.6 (5.7)
Phrase	16	−33.3 (98.6)	4.8 (2.7)	1.1 (2.6)
		**Duration**	**f0**	**Intensity**
T-test between compounds vs. phrases		*t*_(28)_ = 1.11, *p* = 0.27	*t*_(27)_ = 4.61, *p* < 0.001	*t*_(28)_ = 2.93, *p* = 0.007

The mean differences were calculated as follows (a): Duration: the duration of the first syllable vowel (nucleus) of the first part of the compound minus the duration of the first syllable vowel (nucleus) in the second part of the compound or phrase, i.e., “kIssankEllo” or “kIssan kEllo”, respectively. (b) f0 and intensity: the peak value of the f0/intensity in the first part of the compound minus the peak value of the f0/intensity in second part of the compound/phrase. The f0 differences were calculated in semitones. One f0 value was missing due to creaky voice.

**Figure 2:**
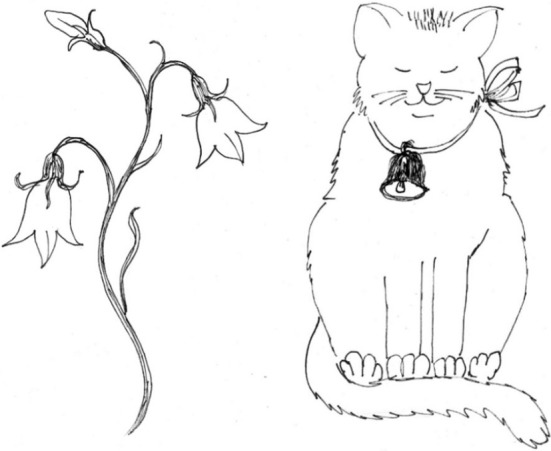
**Example of the word stress task.** The left picture represents a compound word “kissankello” and the right picture a phrase “kissan kello.”

In each trial, the participants heard an utterance produced with a stress pattern that denoted it either as a compound (e.g., “näytä KISsankello” [′kis:an_′_kel:o] meaning “show the harebell flower” or literally “cat's-bell” in English) or as a phrase comprised from the same two words (e.g., “näytä KISsan KELlo” [′kis:an ′kel:o], meaning “show the cat's bell” in English). A similar pair of utterances in English would be, for example, “BLUEbell” and “BLUE BELL”; [′blu_′_bεl] and [′blu ′bεl], respectively. As the participants heard the utterance (supplementary audio files 1 and 2), they were presented two pictures on the screen (see Figure 2) and the task was to choose which picture matched with the utterance they heard by pressing a button. There were six different pairs of utterances (a compound word and a phrase). The utterances were spoken by four different people: an adult male, an adult female, a female child of 10 years and a female child of 7 years. The original Finnish test version used by Torppa et al. (2010) had 48 trials. For the present study a shorter 30 trial version was made by excluding 18 trials of which 2 were found to be too difficult and 16 too easy for the nine healthy adult participants on a pilot study. The duration of the test was ca 4–5 min. The test was carried out using Presentation ® software (www.neurobs.com).

#### Visuospatial perception

**Figure 3:**
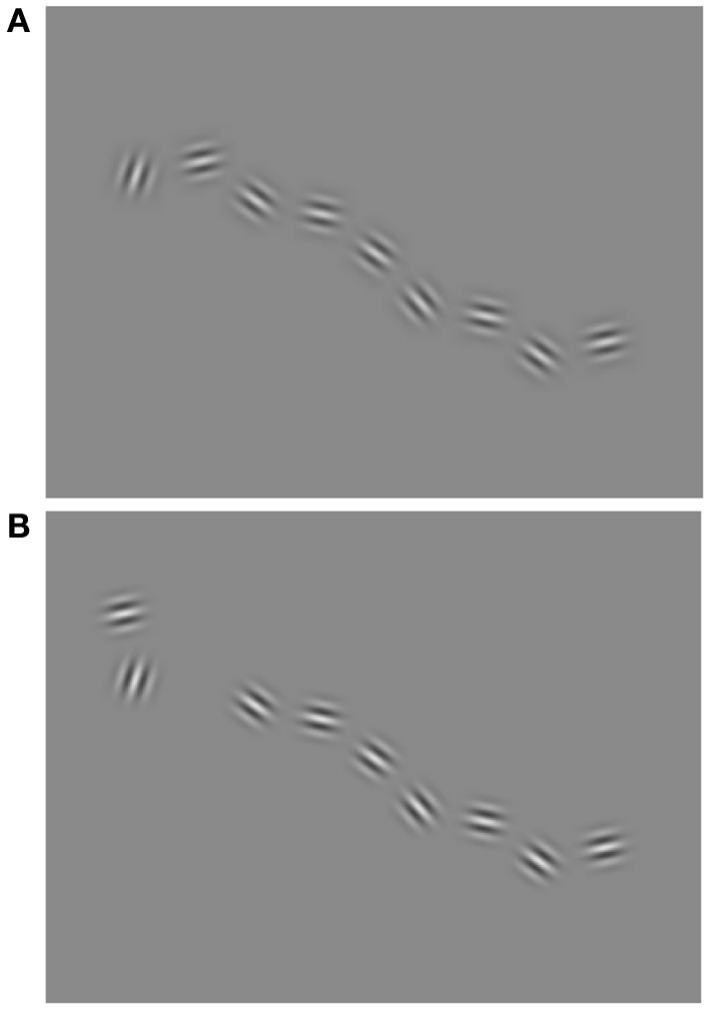
**Example of the visuospatial task with the original sequence of Gabor figures (A) and a sequence with a change in the location and orientation of one of the Gabor figures (B).** Note that in the actual test, only a single Gabor was presented at a time.

Visuospatial perception was assessed by a test that was developed for this study as a visuospatial analogy for the MBEA Scale subtest. The stimuli were created and the test was conducted using Matlab and Psychophysics Toolbox extension (Brainard, 1997). In each trial the participants were presented two series of Gabor patches (contrast 75%; spatial frequency ca. 0.8 c/°; size approximately 2°) proceeding from left to right. There was a 500 ms pause between the two series. A single Gabor was presented at a time (there was a 50 ms pause between two Gabors, the duration of each Gabor varied) and the Gabors formed a continuous path. The path was formed by simultaneously changing the position and the orientation of the Gabor relative to the preceding Gabor. The orientation of the Gabor followed the direction of the path. On half of the trials the two Gabor series were identical, on the other half the second path was changed (Figure 3, Supplementary movie files 1 and 2). In change trials the second series had one Gabor that deviated from the expected path (Figure 3B, supplementary movie file 2). The participants' task was to judge whether the two paths were similar or different. The paths were constructed as analogous to the melodies in the MBEA Scale subtest: each Gabor was analogous to a tone in the melody and each deviating Gabor was analogous to an out-of-scale tone. Every semitone difference in the melody was equivalent to a 12° difference in the Gabor orientation and the corresponding change in Gabor location, except the deviant Gabor that had 22° location change per semitone. The orientation change, 12°, was within the association field of contour integration (Field et al., 1993). Like the MBEA Scale test, the test began with two example trials: one trial with two similar series and one trial with a difference in the second series. The experiment had 30 trials of which 15 contained two similar series and 15 contained a deviant figure in the second series. In a pilot study with 11 participants, the type (location, orientation, both) and the size (4–22°) of the deviant Gabor change were varied. From the different types and sizes the deviant change (location, 22°) was chosen to match the level of difficulty of the MBEA Scale test (Peretz et al., 2003; norms updated in 2008). The duration of the test was ca 10 min.

#### Pitch perception

The pitch perception test was a shortened adaptation of the test used by Hyde and Peretz (2004) and it was carried out using Presentation ® software (www.neurobs.com). In every trial the subjects heard a sequence of five successive tones and their task was to judge if all five tones were similar or if there was a change in pitch. The duration of a tone was always 100 ms and the intertone interval (ITI; onset to onset) was 350 ms. In the standard sequence, all tones were played at the pitch level of C6 (1047 Hz) and in the sequences that contained a change, the fourth tone was altered. The altered tones were 1/16, 1/8, 1/4, 1/2, or 1 semitones (3, 7, 15, 30 or 62 Hz) upward or downward from C6. The different change sizes and changes upward and downward were presented as many times. The order of the trials was randomized. The test contained 80 trials: 40 standard sequences and 40 sequences with the fourth tone altered in pitch. Three example trials are presented in Supplementary files: a standard trial with no change (supplementary audio file 3) and two change trials (1 semitone upwards; audio file 4 and downwards; audio file 5). The test was substantially shorter than the test by Hyde and Peretz (2004). It also contained smaller pitch changes because the difficulty level was set to match the participants who were not recruited because of having problems in the perception of music. The duration of the test was ca 3–4 min.

#### Auditory working memory

Auditory working memory and attention span were measured with the Digit Span subtest of the Wechsler Adult Intelligence Scale III (WAIS-III; Wechsler, 1997). In the first part of the test, the participants hear a sequence of numbers read by the researcher and their task is to repeat the numbers in the same order. In the second part the task is to repeat the number sequence in reverse order. The test proceeds from the shortest sequences (two numbers) to the longer ones (max. nine numbers in the first and eight numbers in the second part of the test). Every sequence that the participant repeats correctly is scored as one point and the maximum total score is 30. The duration of the test was ca 5 min.

#### Questionnaires

The subjects filled out two questionnaires: a computerized questionnaire after the music perception test (same as in Peretz et al., 2008) as well as a paper questionnaire at the end of the assessment session. In the questionnaires the participants were asked about their musical and general educational background; cognitive problems; musical abilities, hobbies, and preferences (see Appendix: Data Sheet 1). The last part of the paper questionnaire was the Brief Music in Mood Regulation -scale (Saarikallio, 2012). The links between music perception, different kinds of musical hobbies and music in mood regulation will be presented elsewhere in more detail: in the present study, only questions regarding first language, cognitive problems, years of musical and general education, and education level, were analyzed.

### Statistical analysis

The associations between the MBEA scores and background variables were first examined using *t*-tests, ANOVAs, and Pearson correlation coefficients depending on the variable type. The variables that had significant associations with the music perception scores were then included in further analysis. Pitch perception and auditory working memory were also regarded as possible confounding variables and controlled for when examining the associations that word stress and visuospatial perception had with music perception. Linear step-wise regression analyses were performed to see how much the different variables could explain the variation of the music perception total score and subtest scores. All statistical analyses were performed using PASW Statistics 18.

## Results

### Descriptive statistics of the MBEA and other tests

**Figure 4:**
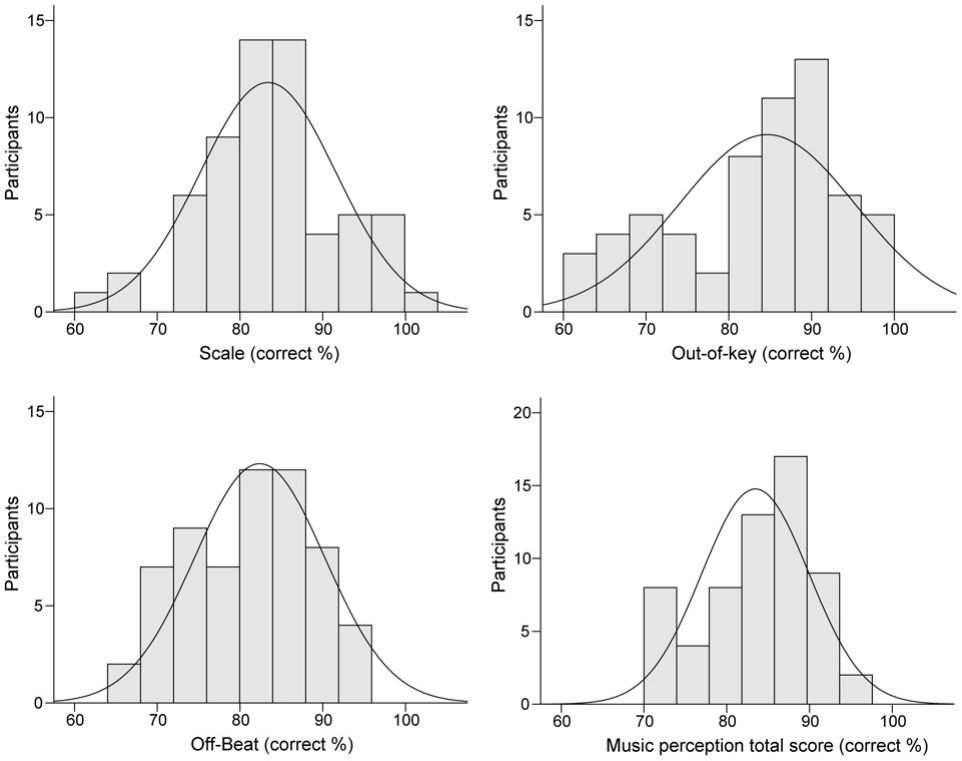
**Distributions of the music perception subtest and total scores**.

**Figure 5:**
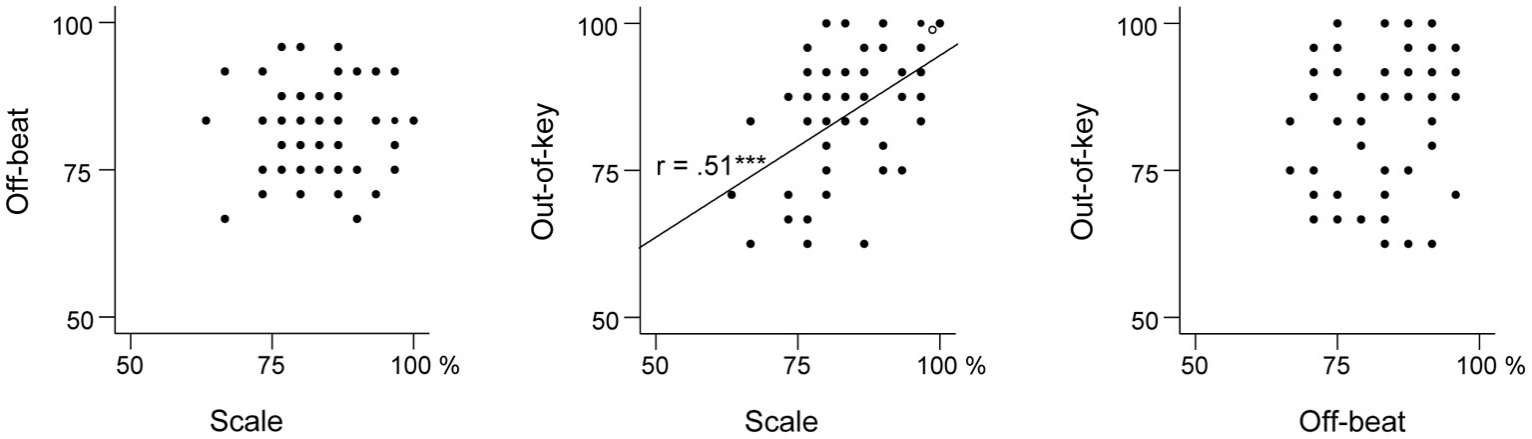
**Scatter plots indicating the relationships between the three music perception subtests**.

Table 3 presents the ranges, means, and standard deviations of the music perception scores. Total music perception scores were calculated as the mean averaged score across the three subtests. Discrimination (d′) and response bias [ln(β)] indexes for the subtests were also calculated. The analysis of d′ yielded highly similar associations to other variables as the proportion of correct answers (hit rate + correct rejections) and hence only the latter is reported. There was no significant correlation between response bias and proportion of correct answers in the music perception total score [*r*_(59)_ = 0.18, *p* = 0.17]. There was a small response bias toward “congruous” responses in the Off-beat [*t*_(60)_ = −15.23, *p* < 0.001] and Out-of-key subtests [*t*_(60)_ = −5.07, *p* < 0.001], and in the total score [*t*_(60)_ = −4.68, *p* < 0.001], but not in Scale subtest [*t*_(60)_ = 1.66, *p* = 0.10]. Based on visual examination, the subtest scores and the total music perception scores were approximately normally distributed (Figure 4). Figure 5 shows the associations between the three music perception subtests. The Scale and the Out-of-key subtests were significantly correlated [*r*_(59)_ = 0.51, *p* < 0.001], whereas Off-beat did not correlate significantly with the other subtests [correlation to Scale *r*_(59)_ = 0.13, *p* = 0.33 and Out-of-key *r*_(59)_ = 0.18, *p* = 0.16].

**Table 3:** **Basic descriptive statistics of the music perception test**.

	**Range**	**Mean**	**Standard deviation**
Scale	19–30 (63.3–100%)	25.0 (83.4%)	3.2 (10.5%)
Off-beat	16–23 (66.7–95.8%)	19.8 (82.4%)	2.4 (10.1%)
Out-of-key	15–24 (63.0–100%)	20.3 (84.6%)	3.3 (13.7%)
Total	55–74 (70.5–94.9%)	65.1 (83.5%)	6.5 (8.3%)

Table 4 shows the ranges, means, and standard distributions of the other tests. Based on visual examination, the scores were approximately normally distributed in all tests. The average performance levels in the word stress (83%) and the visuospatial perception (79%) tasks were almost identical to the average level of performance in the music perception test (84%). The performance in the auditory working memory task was close to the average level in the Finnish population (Wechsler, 2005). In the pitch perception task the largest changes (62 Hz; one semitone) were noticed by all of the participants with 100% accuracy while the smallest changes (3 and 7 Hz) were not noticed at all by some of the participants. Pitch discrimination threshold was calculated as the size of the pitch change that the participant detected with 75% probability.

**Table 4:** **Other tests of perception and memory: basic descriptive statistics**.

	**Range**	**Mean**	**Standard deviation**
Speech prosody perception	19–30 (63–100%)	25.0 (83%)	2.7 (9%)
Visuospatial perception	17–30 (57–100%)	23.8 (79%)	2.9 (10%)
Auditory working memory	10–22 (33–73%)	15.8 (53%)	3.0 (10%)
Pitch perception			
No change trials	13–40 (33–100%)	32.4 (81%)	6.4 (16%)
Change trials	22–38 (55–95%)	30.3 (76%)	4.4 (11%)
3 Hz change (1/16 semitone)	0–7 (0-88%)	2.7 (34%)	2.1 (26%)
7 Hz change (1/8 semitone)	0–8 (0–100%)	4.5 (57%)	2.0 (25%)
15 Hz change (1/4 semitone)	4–8 (50–100%)	7.1 (89%)	0.9 (12%)
36 Hz change (1/2 semitone)	6–8 (75–100%)	7.8 (98%)	0.4 (0%)
62 Hz change (1 semitone)	8 (100%)	8 (100%)	0 (0%)
Pitch discrimination threshold (Hz)	3.0–26.1	9.9	4.7

### Associations between the music perception test and demographical and musical background variables

Gender, first language, self-reported cognitive problems, and self-reported suspected or mild hearing problems were not significantly associated with the music perception total score or any of the subtests (*p* > 0.05 in all *t*-tests). First language was also not significantly associated with the word stress perception, *t*_(59)_ = −1.08, *p* = 0.29. Suspected or mild hearing problems were neither significantly associated with the pitch discrimination threshold [*t*_(59)_ = 0.52, *p* = 0.61] or word stress perception [*t*_(59)_ = 0.55, *p* = 0.59]. The associations to the music perception total score are shown in Table 5. However, owing the relatively small number of the self-reported cognitive problems, possible associations cannot be reliably ruled out for most problems.

**Table 5:** **Background variables' associations with the music perception total score**.

**Background variable**	***N***	**Mean music perception scores (%)**	**Significance of the difference**
Gender: female/male	40/21	84/82	*t*_(59)_ = 0.96, *p* = 0.34
First language: Finnish/Swedish	58/3	84/81	*t*_(59)_ = 0.73, *p* = 0.47
Self-reported cognitive problems			
Problems in reading: yes/no	5/53	83/84	*t*_(56)_ = −0.16, *p* = 0.87
Attention problems: yes/no	5/53	83/84	*t*_(56)_ = −0.30, *p* = 0.76
Problems in speech: yes/no	3/55	79/84	*t*_(57)_ = −1.40, *p* = 0.17
Problems in mathematics: yes/no	12/45	83/84	*t*_(55)_ = −0.69, *p* = 0.49
Memory problems: yes/no	6/51	85/84	*t*_(55)_ = 0.43, *p* = 0.67
Problems in visuospatial orientation: yes/no	5/52	82/84	*t*_(55)_ = −0.79, *p* = 0.43
Suspected or mild hearing problems: yes/no	12/49	81/84	*t*_(60)_ = −1.49, *p* = 0.14

Age was not linearly correlated with the music perception total score [*r*_(59)_ = 0.03, *p* = 0.79], but when the age groups were compared to each other using ANOVA, a significant association was found [*F*_(3, 57)_ = 6.21, *p* = 0.001]. The music perception score seemed to rise until the age group of 40–49 years but the age group of 50–59 years had the lowest scores. *Post hoc* test (Tukey HSD) showed that the age group 40–49 years had significantly higher music perception scores than the groups 19–29 years (*p* = 0.004) and 50–59 years (*p* = 0.002). The average music perception scores of the age groups are shown in Table 6.

**Table 6:** **Average music perception scores of the age groups**.

**Age group (years)**	***N***	**Music perception score mean (sd) (%)**
19–29	17	81.2 (6.2)
30–39	14	85.0 (6.8)
40–49	12	89.0 (3.2)
50–59	18	80.7 (6.5)

Level of education did not differentiate the participants regarding their music perception scores [*F*_(3, 57)_ = 1.81, *p* = 0.16] and neither were education years significantly correlated with music perception [*r*_(56)_ = 0.10, *p* = 0.46]. The participants who had got some kind of music education in addition to the compulsory music lessons in school (*N* = 42) had higher music perception scores than those who only had got the compulsory lessons (*N* = 19) [*t*_(59)_ = 2.75, *p* = 0.008]. The difference was 4.7% on average. The correlation between years of music education (0–19) and the total music perception score was significant [*r*_(51)_ = 0.32, *p* = 0.019].

### Associations between music perception, word stress perception and visuospatial perception

Table 7 shows the correlations between the possible confounding variables (pitch perception, auditory working memory, music education, and general education) and word stress, visuospatial perception, and music perceptions

**Table 7:** **Correlations between speech prosody and visuospatial perception, music perception and possible confounding variables**.

	**Word stress**	**Visuospatial**	**Music perception (total)**
Pitch perception: change trials (*df* = 59)	0.01	0.05	0.31^*^
No change trials (*df* = 59)	−0.06	0.07	−0.15
All trials (*df* = 59)	−0.08	0.14	0.09
Pitch discrimination threshold (*df* = 59)	−0.13	−0.03	−0.32^**^
Auditory working memory (*df* = 59)	0.26^*^	0.10	0.10
Digit span forwards (*df* = 59)	0.26^*^	0.07	0.07
Digin span backwards (*df* = 59)	0.13	0.11	0.06
Music education (years) (*df* = 51)	0.12	0.02	0.32^*^
General education (years) (*df* = 56)	0.08	−0.11	0.10

** p < 0.01;

* p < 0.05.

Step-wise regression analyses were performed to see how much the different variables could explain the variation of the music perception total score and subtests. Four different models of predictors were examined: first the possibly confounding background variables, then the possibly confounding variables measured by tests and lastly the test scores that were the main interest of this study. In the first model, age group (under/over 50 years) and music education (no/yes) were used as predictors. These background variables were included in the regression analysis because they were significantly associated with the music perception scores. Second, pitch discrimination threshold and auditory working memory score were added to the model. Third, visuospatial perception score was added as a predictor. Finally, the word stress score was added to the model. Table 8 shows the regression analyses including the coefficients of determination (*R*^2^) of the different models. As can be seen from the *R*^2^ change in the regression analysis for the total music perception score, both visuospatial perception and word stress perception explained about 8% of the variation of the total music perception score while controlling for music education, age, auditory working memory and pitch discrimination threshold. Music education and pitch discrimination threshold were also significant predictors.

**Table 8:** **Regression analysis**.

**Model**	**Variable**	**Beta**	***T***	***F*(*df*)**	***R*^2^**	***R*^2^ change**
**MUSIC PERCEPTION TOTAL SCORE**						
1				*F*_(2, 58)_ = 5.21^**^	0.15	0.15
	Music education	0.28	2.27^*^			
	Age group	−0.20	−1.62			
2				*F*_(4, 56)_ = 3.77^**^	0.21	0.06
	Music education	0.28	2.25^*^			
	Age group	−0.14	−1.07			
	Auditory working memory	−0.01	−0.05			
	Pitch discrimination threshold	−0.25	−2.07^**^			
3				*F*_(5, 55)_ = 4.43^**^	0.29	0.08
	Music education	0.28	2.37^*^			
	Age group	−0.08	−0.61			
	Auditory working memory	−0.02	−0.19			
	Pitch discrimination threshold	−0.26	−2.23^*^			
	Visuospatial perception	0.28	2.40^*^			
4				*F*_(6, 54)_ = 5.27^***^	0.37	0.08
	Music education	0.28	2.47^*^			
	Age group	−0.09	−0.74			
	Auditory working memory	−0.10	−0.85			
	Pitch discrimination threshold	−0.23	−2.03^*^			
	Visuospatial perception	0.27	2.42^*^			
	Word stress perception	0.30	2.65^*^			
**SCALE SUBTEST**						
1				*F*_(2, 58)_ = 3.67^*^	0.11	0.11
	Music education	0.15	1.20			
	Age group	−0.26	−2.00^*^			
2				*F*_(4, 56)_ = 1.78	0.11	0.00
	Music education	0.15	1.15			
	Age group	−0.25	−1.79^+^			
	Auditory working memory	−0.01	0.10			
	Pitch discrimination threshold	−0.04	−0.30			
3				*F*_(5, 55)_ = 2.05^+^	0.16	0.05
	Music education	0.15	1.19			
	Age group	−0.20	−1.44			
	Auditory working memory	0.00	0.01			
	Pitch discrimination threshold	−0.05	−0.36			
	Visuospatial perception	0.22	1.71^+^			
4				*F*_(6, 54)_ = 1.85	0.17	0.01
	Music education	0.15	1.18			
	Age group	−0.20	−1.47			
	Auditory working memory	−0.03	−0.22			
	Pitch discrimination threshold	−0.03	−0.25			
	Visuospatial perception	0.21	1.67			
	Word stress perception	0.12	0.91			
**OUT-OF-KEY SUBTEST**						
1				*F*_(2, 58)_ = 3.35^*^	0.10	0.10
	Music education	0.31	2.40^*^			
	Age group	−0.04	−0.31			
2				*F*_(4, 56)_ = 2.77^*^	0.17	0.06
	Music education	0.32	2.51^*^			
	Age group	−0.01	−0.04			
	Auditory working memory	−0.12	−0.98			
	Pitch discrimination threshold	−0.23	−1.82^+^			
3				*F*_(5, 55)_ = 2.55^*^	0.19	0.02
	Music education	0.32	2.54^*^			
	Age group	0.03	0.21			
	Auditory working memory	−0.13	−1.06			
	Pitch discrimination threshold	−0.24	−1.87^+^			
	Visuospatial perception	0.15	1.23			
4				*F*_(6, 54)_ = 2.87^*^	0.24	0.05
	Music education	0.32	2.58^*^			
	Age group	0.00	0.00			
	Auditory working memory	−0.20	−1.53			
	Pitch discrimination threshold	−0.21	−1.68^+^			
	Visuospatial perception	0.14	1.18			
	Word stress perception	0.24	1.96^+^			
**OFF-BEAT SUBTEST**						
1				*F*_(2, 58)_ = 1.67	0.05	0.05
	Music education	0.14	1.07			
	Age group	−0.15	−1.15			
2				*F*_(4, 56)_ = 2.83^*^	0.17	0.11
	Music education	0.12	0.90			
	Age group	−0.04	−0.33			
	Auditory working memory	0.13	1.06			
	Pitch discrimination threshold	−0.32	−2.52^*^			
3				*F*_(5, 55)_ = 3.33^*^	0.23	0.06
	Music education	0.11	0.96			
	Age group	0.01	0.10			
	Auditory working memory	0.12	0.97			
	Pitch discrimination threshold	−0.33	−2.67^*^			
	Visuospatial perception	0.26	2.15^*^			
4				*F*_(6, 54)_ = 4.34^**^	0.33	0.09
	Music education	0.12	1.18			
	Age group	0.00	0.00			
	Auditory working memory	0.04	0.32			
	Pitch discrimination threshold	−0.29	−2.48^*^			
	Visuospatial perception	0.25	2.15^*^			
	Word stress perception	0.32	2.73^**^			

*** p < 0.001;

** p < 0.01;

* p < 0.05;

+ p < 0.10.

**Figure 6:**
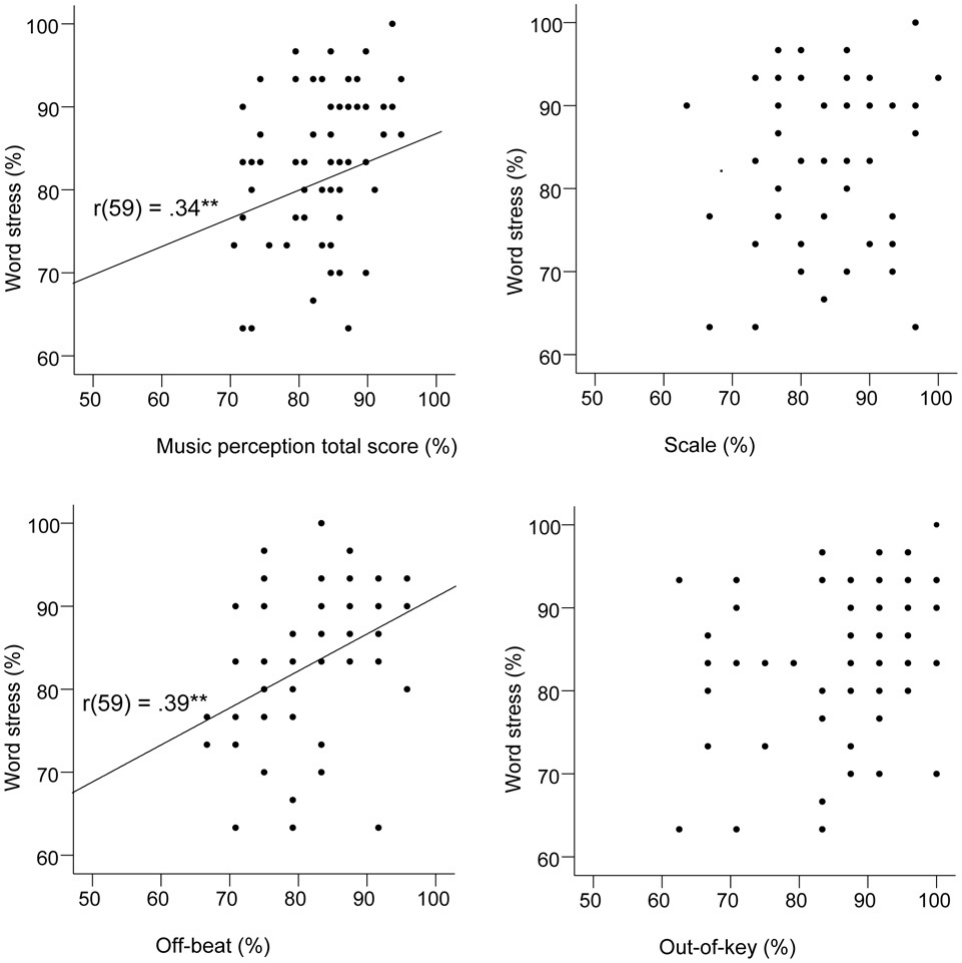
**Scatter plots indicating the relationships between the word stress task and the music perception test**.

When the Scale subtest was analyzed separately, age group was a significant predictor in the first model, but the further regression models were not significant. Visuospatial perception had only a marginally significant association with the Scale subtest that was analogous with it. The final regression model for the Out-of-key subtest was significant and explained 24% of the variance. The most significant predictor was music education. In the regression analysis on the Off-beat subtest, the final model was significant and explained 33% of the variance. The most significant predictor was word stress perception that alone explained 9% of the variance. Figure 6 shows that word stress perception correlated highly significantly with the music perception total score [*r*_(59)_ = 0.34, *p* = 0.007], and with the Off-beat score [*r*_(59)_ = 0.39, *p* = 0.002], but not with the Scale and Out-of-key scores.

## Discussion

The most important finding of this study is the association found between the perception of music and speech prosody, more specifically word stress. Auditory working memory, pitch perception abilities, or background variables like musical education did not explain this association. This finding gives support to the hypothesis that processing music and speech are in some extent based on shared neural resources. The association is found in “normal,” healthy population and thus strengthens the generalizability of the associations previously found in musicians and those having problems in the perception of music or language.

The most powerful background variable influencing the music perception was music education. Age was also found to be related to music perception, as also Peretz et al. (2008) found, but in the present study the association was not linear. Older persons' lower performance in the music perception test might be partly explained by less music education—however, this does not explain the finding that the youngest age group also had lower than average performance. However, the relation between age group and music perception was not very strong, as age group was not a significant predictor in the regression analysis including other more strongly related variables. Gender, general education, and self-reported cognitive problems were not associated with the music perception scores. Music education and age group (under/over 50 years) were controlled for in the statistical analysis and did not affect the associations that were the main findings of this study. Auditory working memory was significantly associated only with the word stress task and did not explain any of the relations that were found.

### Association between music and speech prosody

Patel (2012) argues that the apparent contradiction between the dissociation between speech and music perception found in brain damage studies (Peretz, 1990; Peretz and Kolinsky, 1993; Griffiths et al., 1997; Dalla Bella and Peretz, 1999) and the associations found in brain imaging studies (Patel et al., 1998; Steinhauer et al., 1999; Koelsch et al., 2002; Tillmann et al., 2003; Knösche et al., 2005; Schön et al., 2010; Abrams et al., 2011; Rogalsky et al., 2011) may be explained by a *resource sharing framework*. According to this framework, music and speech have separate representations in long-term memory, and damage to these representations may lead to a specific deficit of musical or linguistic cognition. However, in the normal brain, music and language also share neural resources in similar cognitive operations. In the introduction we also pointed out that the enhanced abilities in music and speech may be based on transfer of training (Besson et al., 2011),—however, as the association found in this study was significant after controlling for musical training, our results may be best interpreted as support for the hypothesis of shared neural resources.

The most important difference between the neural basis of processing speech and music is that at least in most right-handed persons, music is processed dominantly in the right hemisphere while speech is dominantly processed in the left hemisphere (Tervaniemi and Hugdahl, 2003; Zatorre and Gandour, 2008). Zatorre and Gandour (2008) suggest that this difference may not indicate an abstract difference between the domains of speech and music, but it could be explained by the acoustic differences: the left hemisphere is specialized in processing fast temporal acoustic changes that are important for speech perception while fine-grained pitch changes that are important for music perception are more precisely processed by the right hemisphere. However, although the temporal changes that are central in the processing of musical rhythm are relatively slower than those typical in speech, temporal processing is important in processing both speech and musical rhythm. It is thus worth investigating if the processing of musical rhythm may have even more close associations to speech processing than the melodic aspect of music.

#### Rhythm as a new link

Shared neural mechanisms have thus far been found especially concerning pitch perception in music and speech—congenital amusia has been found to be associated with problems in perceiving speech intonation (Patel et al., 2005, 2008b; Jiang et al., 2010; Liu et al., 2010), emotional prosody (Thompson, 2007; Thompson et al., 2012) and the discrimination of lexical tones (Nan et al., 2010). It seems likely that the processing of coarse-grained pitch in music and speech rely on a shared mechanism (Zatorre and Baum, 2012). However, in this study the aim was to find out if music and speech perception may be associated not only regarding the melodic but also the rhythmic aspect. Indeed, the strongest association that the word stress test had with music perception was with the Off-beat subtest that measures the ability to perceive unusual time delays in music. This gives support to the hypothesis that the perception of rhythm in music and speech are connected. Word stress perception was not a significant predictor for performance in the Scale subtest, and for performance in the Out-of-key subtest it was only a marginally significant predictor. The marginally significant relation with the Out-of-key subtest, which measures the ability to notice melodically deviant tones, suggests that melodic cues affect word stress perception in some extent, but as the pitch perception threshold was not associated with the word stress perception scores, it seems likely that the word stress test does not rely on fine-grained pitch perception. This finding suggests that pitch is not the only auditory cue relating the perception of music and speech—the relation can also be found via rhythm perception.

Although previous research has focused more on studying the melodic than the rhythmic aspect of music and speech, there are studies showing that rhythm might actually be a strong link between the two domains. For instance, musicians have been found to perceive the metric structure of words more precisely than non-musicians (Marie et al., 2011b). Also, classical music composed by French and English composers differ in their use of rhythm—the musical rhythm is associated with the rhythm of speech in French and English language (Patel and Daniele, 2003). In a study that investigated the effects of melodic and rhythmic cues in non-fluent aphasics' speech production, the results suggest that rhythm may actually be more crucial than melody (Stahl et al., 2011). Also, children with reading or language problems have been found to have auditory difficulties in rhythmic processing—deficits in phonological processing are associated with problems in the auditory processing of amplitude rise time that is critical for rhythmic timing in both language and music (Corriveau et al., 2007; Corriveau and Goswami, 2009; Goswami et al., 2010; Goswami, 2012). Musical metrical sensitivity has been found to predict phonological awareness and reading development and it has been suggested that dyslexia may have its roots in a temporal processing deficit (Huss et al., 2011). Moreover, the members of the KE family, who suffer from a genetic developmental disorder in speech and language have been found to have problems also in the perception and production of rhythm (Alcock et al., 2000). The affected family members have both expressive and receptive language difficulties and the disorder has been connected to a mutation in the FOXP2 gene that has been considered language-specific (Lai et al., 2001). Alcock et al. (2000) studied the musical abilities of the affected members and found that their perception and production of pitch did not differ from the control group while their rhythmic abilities were significantly lower, suggesting that the disorder might be based on the difficulties of temporal processing. However, as Besson and Schön (2012) point out, genetics can only provide limited information about the modularity of music and language.

Neuroimaging and neuropsychological studies show that musical rhythm is processed more evenly in both hemispheres whereas the melodic aspect is more clearly lateralized to the right hemisphere, at least in most right-handed persons (Peretz and Zatorre, 2005). Because language is more left-lateralized, it is reasonable to hypothesize that it may have shared mechanisms with musical rhythm. Also, perception and production of speech and music have found to be neurally connected (Rauschecker and Scott, 2009)—especially when processing rhythm: motor regions like the cerebellum, the basal ganglia, the supplementary motor area and the premotor cortex are central in the processing of both musical rhythm (Grahn and Brett, 2007; Chen et al., 2008) and speech rhythm (Kotz and Schwartze, 2010). Stewart et al. (2006) proposed that the importance of motor regions give support to a “motor theory” of rhythm perception, as a parallel to the motor theory of speech perception (Liberman and Mattingly, 1985)—the perception of rhythm might be based on the motor mechanisms required for its production. Similar developmental mechanisms for speech and music might explain why training in the other modality causes improvement in the other (Patel, 2008). The association between the perception of rhythm in speech and music can also be related to dynamic attention theory proposing that the allocation of attention depends on synchronization between internal oscillations and external temporal structure (Jones, 1976; Large and Jones, 1999). Recent neuroimaging studies have found evidence that attending to and predictive coding of specific time scales is indeed important in speech perception (Kotz and Schwartze, 2010; Luo and Poeppel, 2012). Kubanek et al. (2013) found that the temporal envelope of speech that is critical for understanding speech is robustly tracked in belt areas at the early stage of the auditory pathway, and the same areas are activated also when processing the temporal envelope of non-speech sounds.

Studies on acquired (Peretz, 1990; Peretz and Kolinsky, 1993; Di Pietro et al., 2004) and congenital amusia (Hyde and Peretz, 2004; Thompson, 2007; Peretz et al., 2008; Phillips-Silver et al., 2011) have found double dissociations between the deficits of melody and rhythm perception in music. Even though congenital amusia is considered to be mainly a deficit of fine-grained pitch perception (Hyde and Peretz, 2004), there are cases of congenital amusia in which the main problem lies in rhythm perception (Peretz et al., 2003; Thompson, 2007; Phillips-Silver et al., 2011). It has been proposed that there are different types of congenital amusia and that speech perception deficits associated with congenital amusia might only concern a subgroup of amusics (Patel et al., 2008b) whereas some might actually have a specific deficit in rhythm perception (Thompson, 2007; Phillips-Silver et al., 2011). In the present study, the Off-beat subtest that measures the perception of rhythmic deviations was not significantly associated with the other music perception subtests measuring the perception of melodic deviations, which further strengthens the hypothesis that rhythm and melody perception can be independent.

Taken together, we found evidence that the perception of speech prosody could be associated with the perception of music via the perception of rhythm, and that the perception of rhythm and melody are separable. This raises the question of whether the type of the acoustic properties (melody or rhythm) of the stimuli under focus might sometimes orient the perception process more than the category (speech or music). This hypothesis is in line with Patel (2012) resource sharing framework suggesting that the cognitive operations might share brain mechanisms while the domains may have separate representations in long-term memory.

### Music and visuospatial perception: is there an association?

Because the perception of musical pitch may be spatial in nature (Rusconi et al., 2006), the possibility of an association between music and visuospatial perception has been suggested. Previous research results considering this association are somewhat contradictory: congenital amusics may have lower than average abilities in visuospatial processing (Douglas and Bilkey, 2007) but this effect has not been replicated (Tillmann et al., 2010; Williamson et al., 2011). In the present study the hypothesis was investigated by creating a visuospatial task that was analogous to the Scale subtest of the MBEA. In the regression analysis where the possible confounding variables were controlled for, the visuospatial test was only marginally significant predictor of the Scale subtest. Also, self-reported problems in visuospatial perception were not found to be significantly associated with the music perception scores. However, visuospatial test was a significant predictor of the music perception total score and Off-beat subtest score when pitch perception, short-term memory, age group, and music education were controlled for. It is possible that these associations may be at least partly explained by some confounding factor (e.g., attention), which we were not able to control for here. Because the expected association between the analogous tests of music and visuospatial perception was not significant, the present results remain somewhat inconclusive concerning the link between music perception and visuospatial processing, and more research is still needed.

## Conclusions

The main result of this study is the observed strong association between music and word stress perception in healthy subjects. Our findings strengthen the hypothesis that music and speech perception are linked and show that this link does not exist only via the perception of pitch, as found in former studies, but also via the rhythmic aspect. The study also replicated former findings of the independence of rhythm and melody perception. Taken together, our results raise an interesting possibility that the perception of rhythm and melody could be more clearly separable than music and speech, at least in some cases. However, our data is not able to provide any definitive answers and it is clear that more work is still needed.

In future, more research is still needed to better understand the association between the processing of rhythm or meter in speech and music. The perception of rhythm comprises of many aspects and it is important to find out exactly which characteristics of the rhythm of speech and music are processed similarly. One possibility is to study communication forms in which rhythm is a central variable for both speech and music—rap music might be one example. This line of research would be interesting and fruitful in delineating the commonalities and cross-boundaries between music and speech in the temporal and spectral domains.

### Conflict of interest statement

The authors declare that the research was conducted in the absence of any commercial or financial relationships that could be construed as a potential conflict of interest.
