# Supplementary material for: Music and speech prosody: a common rhythm
Source: Front Psychol. 2013 Sep 2;4:566. doi: 10.3389/fpsyg.2013.00566 (PMC3759063; doi:10.3389/fpsyg.2013.00566)
Supplement: Supplementary file 3 [file DataSheet1.DOCX]

**Questionnaire 1. (computerized, along with the music perception test)**

All information collected here will remain confidential. The questions preceded by a star (*) are obligatory.

Personal Information

1. Your full name

2. Telephone number and/or email

* 3. Birth date

* 4. Gender (male – female)

**Educational and professional background**

1. Education

* Level of education

Primary school – secondary school – lowest tertiary – Bachelor’s degree – Master’s degree - PhD

* Number of years of education (since kindergarten)

2. Profession

3. Do you experience (or have you ever experienced) problems in any of the following areas

yes no

dyslexia

attention

memory

speech

mathematics

spatial orientation

other (please specify)

**Music in your everyday**

1. Do you intentionally listen to music?

Never – rarely – sometimes – often – very often

2. Do you sing in private (in the car, in the shower)?

Never – rarely – sometimes – often – very often

3. Do you sing in public (with friends, in a choir, karaoke, etc.)?

Never – rarely – sometimes – often – very often

4. Do you dance?

Never – rarely – sometimes – often – very often

If so, would you consider yourself to be a good dancer?

Terrible – not very good – moderate – good – excellent

* 5. Can you recognize a very familiar melody (such as the national anthem) without the help of lyrics?

Never – rarely – sometimes – often – very often

6. For each phrase, please indicate which one(s) corresponds to you.

Yes No

I love music, listening to music is a real pleasure

Music is like a foreign language to me

I listen to music, but I can go without it; music is not very important to me.

**Your musical ear**

* 1. Do you think that you lack a sense of music? (yes – no)

2. How do you know that you lack a sense of music?

* 3. Can you perceive when someone sings out-of-tune? (yes – no)

* 4. Can you perceive when someone produces a wrong note? (yes – no)

5. Please check which of the following statements best correspond to your situation.

yes no

I sing out-of-tune

I cannot dance

I cannot remember songs or melodies

I cannot sing back notes after they have been played on the piano

a parent, peer or teacher told me that I was tone-deaf

I cannot follow a musical rhythm

6. If I sing...

I can tell when I am singing out-of-tune and can correct myself

I can tell when I am singing out-of-tune but cannot correct myself

I cannot tell when I am singing out-of-tune, unless someone else tells me

I do not sing

**Music in your childhood**

When you were a child (before 11 years of age), what was your surrounding musical environment (family, cultural environment, school, etc.)?

1. Was listening to music an important part of your environment?

Never – rarely – sometimes – often – very often

2. Did your mother sing to you when you were growing up (e.g. lullabies)?

Never – rarely – sometimes – often – very often

4. Did your mother have any musical problems?

Yes – no – don’t know

5. Did your father have any musical problems?

Yes – no – don’t know

6. Do you think any of your siblings have any musical problems (singing out-of-tune, cannot recognize familiar songs, no sense of rhythm, do not appreciate or do not enjoy listening to music)?

brother(s)

brother(s) with difficulties

sister(s)

sister(s) with difficulties

**Your musical education**

1. What type of musical education have you received?

yes no

none

self-taught

obligatory music class at school

optional music class at school

private music lessons, or with your parents

musical conservatory

If you answered 'none', please pass directly to section 07 (Further Information)

2. At what age did you begin music lessons? (excluding the obligatory school courses)

years old

3. For how long did you take music lessons?

years old

4. To what degree was this experience frustrating

Not at all – somewhat – moderately – very much – extremely

5. Do you still sing or play this same instrument? (yes – no)

**Further Information**

1. Do you have (or have you had) any health problems such as:

yes no If so, when?

a cerebral vascular accident

a brain trauma

2. Have you ever seen an audiologist? (yes – no)

If so, why and when? What was the diagnosis?

3. Are you color blind? (yes – no)

4. Are there other color blind person in your family? (yes – no)

**Contact Information**

1. Would you be interested in participating in future studies? (yes – no)

2. How may we best contact you?

mail

civic address

city or town

postal code/ZIP

state or province

country

**Questionnaire 2. (on paper, at the end of the test session)**

**A) Background**

1. Initials _______
2. Age___
3. Gender: a) Female b) Male
4. First language

a) Finnish

b) Some other language, what?_________

If your first language is other than Finnish, are your skills in Finnish comparable to first language level? c) Yes d) No

**B) Musical Background**

1. Before school age, did you go to musical playschool?

a) Yes b) No

1. Have you been on a special music class in school

a) Yes

- grades 1-6

- grades 7-9

- secondary school

b) No

**C) Music-related hobbies**

1. How often do you listen to music actively (without doing anything else at the same time)?

Not at all – once a year – once a month – 2–3 times a month – once a week – daily

1. How often do you listen to music passively (e.g. when you are cleaning)?

Not at all – once a year – once a month – 2–3 times a month – once a week – daily

1. How often do you go to concerts?

Not at all – once a year – once a month – 2–3 times a month – once a week – daily

1. How often do you play an instrument?

Not at all – once a year – once a month – 2–3 times a month – once a week – daily

1. How often do you sing?

Not at all – once a year – once a month – 2–3 times a month – once a week – daily

1. How often do you dance or do physical exercise with music (e.g. zumba)?

Not at all – once a year – once a month – 2–3 times a month – once a week – daily

1. Please estimate the importance of music in your daily life:

Not at all important 1 ----- 2 ----- 3 ----- 4 ----- 5 very important

**D) Other hobbies**

1. How often do you go to theater, movies, museums or art exhibitions?

Not at all – once a year – once a month – 2–3 times a month – once a week – daily

1. How often do you meet friends or take part in social or organizational activities?

Not at all – once a year – once a month – 2–3 times a month – once a week – daily

1. How often do you play games (e.g. sudoku, computer games, board games)?

Not at all – once a year – once a month – 2–3 times a month – once a week – daily

1. How often do you read?

Not at all – once a year – once a month – 2–3 times a month – once a week – daily

1. How often do you exercise?

Not at all – once a year – once a month – 2–3 times a month – once a week – daily

1. How often do you engage in artistic activity yourself (e.g. theater, visual arts, photography)?

Not at all – once a year – once a month – 2–3 times a month – once a week – daily

**E) Musical preferences**

1. Please estimate how much do you like to listen to following music styles. Mark one of the numbers on the line after the music style.

do not like at all 1 ------- 2 -------- 3 -------- 4 --------- 5 like very much

Classical: _______

Jazz: _______

Rock: _______

Pop: _______

Easy listening: ________

Something else, what: ______________ ________

**F) Music in mood regulation (B-MMR; see Saarikallio, 2012)**
